# Characterization of G-Quadruplexes Folding/Unfolding Dynamics and Interactions with Proteins from Single-Molecule Force Spectroscopy

**DOI:** 10.3390/biom11111579

**Published:** 2021-10-25

**Authors:** Yuanlei Cheng, Yashuo Zhang, Huijuan You

**Affiliations:** Hubei Key Laboratory of Natural Medicinal Chemistry and Resource Evaluation, School of Pharmacy, Tongji Medical College, Huazhong University of Science and Technology, Wuhan 430030, China; D201981435@hust.edu.cn (Y.C.); D202181686@hust.edu.cn (Y.Z.)

**Keywords:** single-molecule manipulations, G-quadruplex, polymorphism, kinetics, mechanical stability, G4 helicase, nucleic acid chaperone

## Abstract

G-quadruplexes (G4s) are stable secondary nucleic acid structures that play crucial roles in many fundamental biological processes. The folding/unfolding dynamics of G4 structures are associated with the replication and transcription regulation functions of G4s. However, many DNA G4 sequences can adopt a variety of topologies and have complex folding/unfolding dynamics. Determining the dynamics of G4s and their regulation by proteins remains challenging due to the coexistence of multiple structures in a heterogeneous sample. Here, in this mini-review, we introduce the application of single-molecule force-spectroscopy methods, such as magnetic tweezers, optical tweezers, and atomic force microscopy, to characterize the polymorphism and folding/unfolding dynamics of G4s. We also briefly introduce recent studies using single-molecule force spectroscopy to study the molecular mechanisms of G4-interacting proteins.

## 1. Introduction

**Figure 1:**
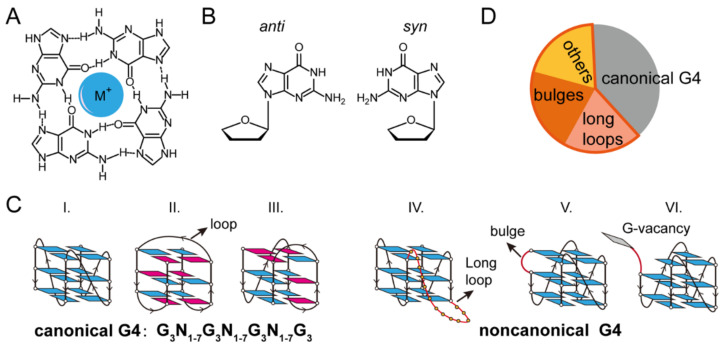
The structure of G-quadruplexes. (**A**) A G-quartet is associated with four guanines and stabilized by Hoogsteen hydrogen bonding and monovalent cations. (**B**) The *anti* and *syn* glycosidic bonds. (**C**) Schematic representation of canonical and noncanonical G4 topologies. Canonical G4s can be classified as parallel (four strands with the same orientation, I), antiparallel (four strands opposite to each other, II), and hybrid-stranded (one strand with the opposite orientation to the other three strands, III). Noncanonical G4s bear some special structural features, such as the G4 with a long loop (IV), G4 with a bulge (V), and G4 with a guanine vacancy (VI). (**D**) Populations of G4 structures in the human genome using high-throughput sequencing methods [24].

Guanine-enriched DNA and RNA nucleic acids have the potential to fold into inter- or intramolecular secondary structures known as G-quadruplexes [1,2,3,4,5]. In 1962, Gellert et al., using X-ray crystal diffraction, discovered that guanine-rich DNA can stack into cyclic arrangements via Hoogesteen hydrogen bonds (Figure 1A) [1]. G4 structures are further stabilized by monovalent cations such as K^+^ or Na^+^ in physiological conditions [6]. The potential G4-forming sequences are enriched in telomere regions, promoter regions, DNA replication origins, as well as transcription start sites and play crucial roles in fundamental biological processes [2,3]. The breakthrough proved that the in vivo existence of G4s in human cells came from the development of G4-specific antibodies BG4 [7,8] and 1H6 [9], which allow the visualization of G4s in the fixed human cell [7] and genome mapping of G4s by pull-down experiments [8]. By expressing a small G4 probe protein (6.7 kDa) in cells, a recent study has detected G4 DNA in living cells, and their results showed that >60% of promoters and ~70% human genes contain G4s [10]. G4 structures have attracted much attention because small molecules target G4s in the human telomere regions and proto-oncogene promoter regions or induce G4-related DNA damage; thus, they are potential anti-cancer therapeutic strategies [11,12,13,14,15,16]. G4s also exist in multi-species, such as bacteria, viruses, fungi, and parasites, which indicates that G4s in pathogens may act as specific targets for disease therapies [17,18].

G4s exhibit high conformational polymorphism and can adopt diverse folding topologies, which are characterized by the *syn-* or *anti*-conformation of glycosidic bonds, the strand orientation of the G-tetrad core (parallel, antiparallel, and hybrid), the number of stacking G-quartets, and the loop regions (Figure 1B,C) [19,20]. Bioinformatics analyses using the consensus sequence formula G_≥3_N_1–7_G_≥3_N_1–7_G_≥3_N_1–7_G_≥3_ revealed that there are more than 300,000 canonical G4-forming DNA sequences in the human genome, where G refers to adjacent guanines, and N refers to any base in the loop region [21,22]. An updated overview of the current computational approach to predict G4-forming sequences has been reviewed recently [23]. These sequences form various G4 conformations, and a G4-forming sequence can adopt multiple conformations coexisting in a solution. The G4 conformations depend on the length of the loops and the composition of the sequences, as well as the environmental conditions (e.g., ion condition, molecular crowding). Recently, the definition of G4 structures has been broadened by high-throughput sequencing experiments, which have reported more than 700,000 potential G4-forming sequences in the human genome, including a significant number of noncanonical G4s, such as long-looped G4s, bulged-G4s, and guanine-vacancy-bearing G4s (Figure 1D) [24]. Characterization of the polymorphism and the folding/unfolding dynamics are essential for understanding the regulatory role of G4s in vivo and are also important for using G4s in DNA nanotechnology, such as G4-based DNAzymes, biosensors, and aptamers [25].

The conformational polymorphism and folding/unfolding dynamics of G4 structures have been studied using ensemble average methods, such as NMR [19,20,26], circular dichroism (CD) [27,28,29,30,31], UV absorption spectrum [28,30], and fluorescence resonance energy transfer (FRET) [32,33]. The folding rates of intermolecular G4 formation are very low and often require high DNA concentrations (µM to mM) to form in vitro. In contrast, intramolecular G4s formed by four consecutive G-tracts are often folded in seconds or minutes; thus, they are more biologically relevant. Many excellent reviews have described the thermodynamic and kinetic properties of G4s obtained by ensemble methods [34,35,36]. Computational and theoretical approaches such as molecular dynamic simulations can provide insights of G4s folding/unfolding dynamics at atomistic resolution [37,38,39]. However, due to the coexistence of multiple G4 conformations in solutions, it remains challenging to resolve the dynamic properties of these G4 structures by bulky biochemical methods. The computational approaches need to be verified by experimental evidence [39].

Single-molecule approaches including force-based and fluorescence-based techniques enable the detection of folding/unfolding dynamics in real time and the discovery of diverse G4 topologies in a heterogeneous sample [40,41,42,43,44,45]. Force-based techniques (also called single-molecule force spectroscopy), such as optical tweezers (OT), magnetic tweezers (MT), and atomic force microscopy (AFM), allow direct manipulation of single molecules of DNA, RNA, and proteins. These single-molecule force–spectroscopy techniques have deepened our understanding of the mechanical properties of biomolecules [46], folding/unfolding dynamics [47,48], and the interactions between biological macromolecules [49]. In the past two decades, the folding/unfolding dynamics of G4s have been studied by OT [50,51,52,53,54], AFM [55,56,57,58], and MT [59,60,61,62]. In vivo, G4s are subjected to various mechanical modifications during transcription and replication, and motor proteins such as polymerases or helicases may exert forces on G4s. Therefore, evaluating force-dependent folding/unfolding kinetics and the activities of G4s relating to enzymes are essential to understand the regulatory functions of G4s. Herein, this mini-review mainly focuses on the application of single-molecule force spectroscopy to study the conformational polymorphism, the folding/unfolding kinetics of G4s, the molecular mechanisms of G4s and proteins such as helicase DHX36 (also known as RHAU or G4R1) [63], the G4-specific antibody BG4 [64], and the effects of G4s on the processivity of telomerase [65]. Fluorescence-based techniques, such as single-molecule FRET (smFRET) [42,43,66], are also powerful tools for study of the dynamics of G4s [32,67,68], interactions of G4s with proteins [43,44,69,70,71,72,73,74,75,76,77], and visualization of G4s dynamics in living cells [78]. Several studies combined smFRET and force–spectroscopy to provide more comprehensive information of G4s structural diversity in the presence of external forces [54,60,79]. In this mini-review, we will briefly compare the results obtained by force and fluorescence-based techniques, but we do not discuss the methodology details of smFRET as this has been discussed in recent excellent reviews [42,43,66].

## 2. Single-Molecule Force Spectroscopy for the Investigation of G-Quadruplexes

### 2.1. Single-Molecule Force Spectroscopy

In single-molecule manipulation experiments, mechanical tension (0.01 to 10^4^ pN) is applied to a single biological macromolecule using a force probe, and the structural transition of the molecule associated with extension changes along the stretching direction is monitored (spatial resolution of 0.1 to 10 nm) in real time [80]. OT experiments use dielectric particles such as polystyrene beads (usually with a diameter of 0.5~2 µm) as a force probe and use focused laser beams to exert force on the beads. By moving the trap center, the DNA is stretched to a known extension. The force in OT is measured based on the displacement of the bead from the trap center and the stiffness of the optical trap using Hooke’s law, *F* = −*k*x, where *k* is stiffness of the trap. AFM experiments use a sharpened needle tip (radius approximately 10 nm) fixed on a micro-fabricated cantilever (20 to 300 µm in length) as a force probe. The force (pN to nN) is applied to the molecules immobilized on the tip through vertically moving the cantilever by piezoelectric actuators. The deflection of the cantilever is captured from the displacement of a detection laser reflected on a position-sensitive detector. MT experiments use a superparamagnetic bead (usually with a diameter of 1~3 µm) as a force probe. The force is applied by a gradient magnetic field produced by permanent magnets or electromagnetic fields. By moving the magnet, the force applied to the molecule is changed, and the bead height change is monitored based on the diffraction pattern of the image of the beads using an optical microscope with a CCD camera [81]. At a constant force, the bead height change in MT measurements is only from the extension change of tethered molecules. The instrumentation and the comparison of the spatial resolution of various force-spectroscopy methods are available in a systematic review [80].

It should be noted that there are two types of mechanical constraints: the constraint of position (namely R-constraint) and the constraint of force (namely F-constraint). In R-constraint measurements, the position of a Hookian spring (optical trap center or the position of the AFM cantilever) attached to the tethered molecule is held at constant, and the force is measured as a function of extension. In F-constraint measurements, which are implemented in MT or force-clamping AFM and OT, the extension is recorded as the force is held at constant. Compared to force-clamping AFM and OT, MT can apply a constant force without the implementation of a feedback control, therefore offering good force stability over a long-time period.

**Figure 2:**
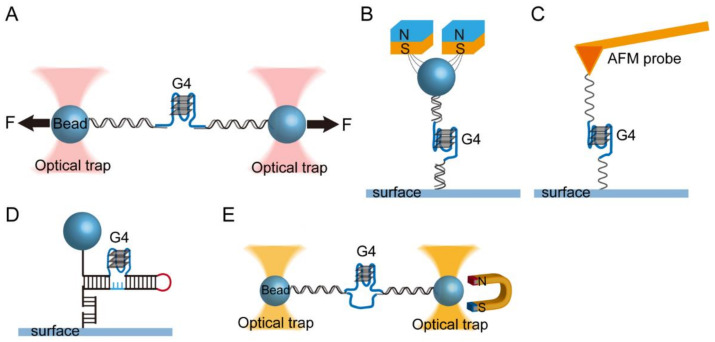
(**A**–**C**) Schematics of single-molecule force spectroscopy assays. Optical tweezers (OT) (**A**), magnetic tweezers (MT) (**B**), and atomic force spectroscopy (AFM) (**C**). A single-stranded G4-forming DNA or RNA sequence (blue) is tethered by dsDNA (**A**,**B**) or ssDNA (**C**) handles (black). (Adapted with permission from [50], American Chemical Society; [61], Oxford Academic; [87], American Chemical Society). (**D**,**E**) G4s embedded in double-stranded DNA studied by MT (**D**) and magneto-optical tweezers (**E**). (Adapted with permission from [90], Oxford Academic; [88], American Chemical Society).

For measuring the folding/unfolding structural transition of intramolecular G4s, a single-stranded DNA consisting of a quadruplex-forming G-rich sequence is ligated with dsDNA handles and tethered between a surface and a force probe (Figure 2A,B) [50,59,61]. The two ends of the DNA are labeled with biotin and digoxigenin or other tags (e.g., thiol, amine) to connect with force probe through biotin-streptavidin and dig-anti-dig or covalent bonding. This design can be used to study the mechanical stability of RNA G4s, such as human telomeric RNA (TERRA), where the single-strand RNA G4-forming sequence is incorporated between two double-stranded DNA/RNA hybrid spacers [82,83]. Click chemistry has also been used to connect the handles to different positions of G4s [84,85,86]. In AFM measurements, a G4-forming ssDNA with two single-strand DNA linkers labeled with 5′-biotin and 3′-thiol can also be directly tethered between the surface and AFM tip (Figure 2C) [87]. In vivo, many DNA G4-forming sequences are embedded in dsDNA. To address the formation of G4s in double-stranded DNA, several studies have used G4-forming sequences embedded in double-stranded DNA [56,88,89] or long DNA hairpins [90] (Figure 2D,E). Together with the DNA origami technique, the constructed DNA tether in force spectroscopy can contain a G4 structure placed inside a DNA origami nanocage that allows quantification of the folding kinetics of G4s in a confined space [91,92,93].

### 2.2. Thermodynamics and Kinetics of G4s Obtained from Single-Molecule Force Spectroscopy

To understand how nucleic acids and proteins fold in response to mechanical forces, it is essential to understand the fundamental biophysical principles such as energy landscape theory. A more general description of the basic thermodynamic and kinetic is available in a previous outstanding review [47]. In this section, we describe how to use two widely used manipulation methods, the force-clamp and force-ramp measurements, to obtain the force-dependent folding and unfolding kinetics of G4s.

**Figure 3:**
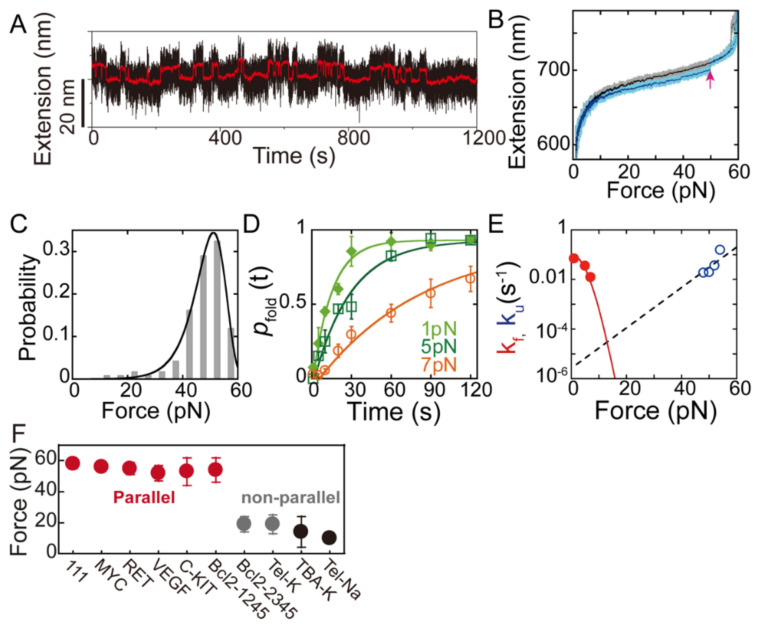
Folding/unfolding kinetics of G4s obtained from force–spectroscopy measurements. (**A**) Force-clamp experiments. Extension of a telomeric G4s in 100 mM KCl buffer held at a constant force of 5 pN fluctuated between folded (low extension) and unfolded (high extension). The red lines show smoothed time traces. The extension record yielded the probability distribution p(x) and the lifetime of folded and unfolded states. (Adapted with permission from [61], Oxford Academic). (**B**) Force-ramp measurement. A representative trajectory of a c-Myc G4s was obtained by a linearly increased force of 0.2 pN/s (cyan). The sudden extension jump (red arrow) is the G4 unfolding signal. (**C**) Unfolding force histogram of Myc2345 G4s. (**D**) The time-evolution of folding probability measured at different forces. (**E**) Force-dependent folding and unfolding rates of Myc2345 G4s. The *k*_f_ was fitted based on the Arrhenius law and force response of ssDNA and G4s. The *k*_u_ was fitted using Bell’s model. ((**B**–**E**), adapted with permission from [94], American Chemical Society). (**F**) The major unfolding force peak of representative G4s. (Adapted with permission from [98], American Chemical Society).

In force-clamp measurements, the reversible folding and unfolding transition of G4 molecules associated with an extension change is recorded at constant force to directly obtain the probability distribution of the molecule extension $P\left(x\right)$ and the folding and unfolding kinetics (*k*_f_ and *k*_u_) (Figure 3A). Boltzmann’s law allows us to calculate how force affects the equilibrium between two or more structural states (e.g., folded G4s and unfolded ssDNA) at a constant force *F*:

(1) $$\Delta G\left(F\right)=-k_{B}T\times ln\left[K_{eq}\left(F\right)\right]$$

where $\Delta G\left(F\right)$ is the free energy difference of folded and unfolded states, *k*_*B*_ is Boltzmann’s constant, *T* is absolute temperature, and *K*_*eq*_ is the equilibrium constant at force *F* (*K*_*eq*_ = *P*(x_2_)/*P*(x_1_)). The effects of force on the unfolding free energy can be calculated as

(2) $$\Delta G\left(F\right)=\Delta G^{0}-\int_{0}^{F}\Delta x\left(F^{\prime }\right)dF^{\prime }$$

where $\Delta G^{0}$ is the unfolding free energy of the structure in the absence of external force. Δx(*F*) is the extension difference of the molecule in unfolded ssDNA and folded G4s, which can be derived based on the force–extension curves of ssDNA and G4s [94]. The force–extension curve of ssDNA is effectively described by the worm-like chain model of polymer elasticity [95]. At the critical force of *F*, the molecule has an equal probability of having folded and unfolded states ($\Delta G\left(F^{*}\right)=-k_{B}T\times ln\left[K_{eq}\left(F^{*}\right)\right]=0$); thus, the zero-force unfolding free energy can be directly estimated based on the critical force *F*:

(3) $$\Delta G^{0}=\int_{0}^{F^{*}}\Delta x\left(F^{\prime }\right)dF^{\prime }$$

The force-dependent folding and unfolding kinetics of G4s at constant force can be obtained based on an analysis of the lifetime of folded and unfolded states [60,61]. The force-dependent folding and unfolding rates are governed by the Arrhenius law:

(4) $$k\left(F\right)=k^{0}\exp\left(\frac{\Delta G^{‡}}{k_{B}T}\right)$$

where $\Delta G^{‡}\left(F\right)=-\int_{0}^{F}\Delta x\left(F^{\prime }\right)dF^{\prime }$ is the additional free energy difference between the transition state and folded state (or unfolded state) caused by force. Bell’s model suggests that the energy barrier lowering by the effect of force *F*Δ*x*_u_, when the unfolding transition distance Δ*x*_u_ is a constant [96]. Both G4s and the transition state have compact structure; therefore, the constant approximation of Δ*x*_u_ is valid, $k_{u}\left(F\right)=k_{u}^{0}\exp\left(\frac{\Delta x_{u}F}{k_{B}T}\right)$. For the folding transition, the elasticity of ssDNA suggests a strong force dependence of the folding transition distance Δ*x*_f_, especially at the low force region, thus, force–extension curve of ssDNA was used to calculate $\Delta G^{‡}\left(F\right)$ for folding transition [94]. For the two-state folding/unfolding transition, the force-dependent free energy difference between the unfolded and folded states can be obtained from the *k*_u_ and *k*_f_ by

(5) $$\Delta G\left(F\right)=-k_{B}T\times \ln\frac{k_{u}\left(F\right)}{k_{f}\left(F\right)}$$

An example of using constant force measurements is through the combination of MT and smFRET used to study the reversible folding/unfolding transition of telomere DNA G4s in the 100 mM Na^+^ solution at near the critical force *F* = 2.5 pN [60]. The force-dependent unfolding rates of telomeric G4s suggest a short unfolding transition distance (Δ*x*_u_~0.6 nm). For the telomeric G4-forming sequence in a physiologically relevant K^+^ solution, the lifetime analysis suggests a complicated folding pathway of G4s including an ultra-stable state that has an extremely long lifetime [61]. Moreover, the equilibrium folding/unfolding transition is not always attained in experiments. For example, parallel-stranded G4s formed by oncogene promoter sequences such as c-Myc has an extremely slow transition rate near the critical forces, making it challenging to determine the folding/unfolding kinetics based on equilibrium measurements [94].

Compared to force-clamp experiments, the force-ramp experiments measure the unfolding force distributions in repeating force-ramp cycles with linearly increased forces (constant loading rate), thus allowing measurement of the unfolding forces of stable G4s (*k*_u_~10^−6^ s^−1^). Another advantage in force-ramp experiments is that for sequences that form multiple structures, the distribution of the unfolding forces and unfolding step sizes can provide information of polymorphism of G4s [40,62,79]. Figure 3B shows an example of force-ramp experiments using MT by changing the magnetic field with time. The DNA constructs containing G4-forming ssDNA were held at a low force for a certain time to allow G4 refolding, and then the force increased linearly to ~60 pN with a loading rate of 0.2 pN/s. A sudden extension jump with a step size of ~7 nm in the force–extension curve represents the G4s unfolding signal. By repeating the force-ramp process, the unfolding force distributions of G4s can be obtained (Figure 3C). To obtain the unfolding rates of the G4s, we fitted the unfolding force distributions to Bell’s model [96]:

(6) $$p\left(f\right)=\frac{k_{u}^{0}}{r}exp\left\{\frac{\Delta x_{u}F}{k_{B}T}+\frac{k_{B}Tk_{u}^{0}}{\Delta x_{u}F}\left[1-exp\left(\frac{\Delta x_{u}F}{k_{B}T}\right)\right]\right\}$$

where *r* is the loading rate, $k_{u}^{0}$ is the zero-force unfolding rate, and Δ*x*_*u*_ is the unfolding transition distance. Multiple single-molecule studies of various G4 structures all suggested a short unfolding transition distance of G4s (<2 nm) [97,98]. The Dudko model has also been used to analyze the unfolding force distributions as it can provide information on the energy barrier [79,97]. In force-ramp experiments, the folding rate *k*_f_ of G4s can be obtained by measuring the time-evolution of the refolding probability *p*_fold_(*t*) and fitting *p*_fold_(*t*) with a single exponential function *p*_fold_ (*t*) = *p*_st_ [1 − exp(−*k*_f_ *t*)] (Figure 3D,E) [50]. The free energy can be also estimated using non-equilibrium theorems such as Jarzynski equality [40,50,99,100]; however, such an approach tends to overestimate the unfolding free energy, particularly when the experiments are far from the equilibrium.

### 2.3. Folding/Unfolding Dynamics and Polymorphism of G4s

In 2009, Yu et al. reported a pioneer work of the mechanical stability of insulin-linked polymorphic region (ILPR) promoter G4s, in which they used optical tweezers and force-ramp experiments at a loading rate of 5.5 pN/s and physiologically relevant concentration of 100 mM K^+^. The unfolding force histogram revealed two populations with the force centered at 22.6 and 36.9 pN, which represent parallel and antiparallel G4 structures [50]. By measuring the time-evolution folding probability, Yu et al. also discovered that the folding rates *k*_f_ were 0.4 vs. 0.3 s^−1^ for parallel and antiparallel G4 structures, respectively [50]. The effects of the loading rates on ILPR G4s unfolding have been characterized by Messieres et al. [97]. They employed optical tweezers to measure the unfolding forces of ILPR G4s at different loading rates (2.1, 7.0, and 23.9 pN/s) to obtain the unfolding energy barrier and the zero-force unfolding rate of G4s. The unfolding force peaks are insensitive to loading rates, which suggests a short unfolding transition distance.

Since then, many G4-forming sequences have been measured, and the telomere G4-forming single-stranded DNA (TTAGGG)_4_ is the most characterized [52,54,59,60,61,79]. By combining single-molecule FRET and magnetic tweezers, Long et al. first characterized the equilibrium folding/unfolding transition of telomere G4s in the presence of a 100 mM Na^+^ solution [60]. Due to the critical force for telomere DNA G4s in Na^+^ solution being *F*~2.5 pN and the extension changes due to unfolding transition being~2.1 nm at 2.5 pN, the smFRET signal was used to quantify the lifetime of folded and unfolded states under tension (τ_folded_~4.1 s and τ_unfolded_~0.76 s). In contrast, telomere G4s in the presence of 100 mM KCl showed a reversible folding/unfolding transition near the critical force *F* of 5–7 pN, which is consistent with the higher thermodynamic stability of telomere G4s in K^+^ than Na^+^ solution [61]. Based on the lifetime analysis of telomere G4s held at 5 pN, You et al. reported three folded states of telomere G4s: a short-lived intermediate state (lifetime~3 s), a long-lived intermediate state (lifetime~45 s), and an ultra-long-lived state (lifetime > 1000 s), in the presence of physiologically relevant 100 mM K^+^ [61]. This result is consistent with the multiple states observed in a previous smFRET study [67] but at the much lower concentration of 10 mM K^+^. The major unfolding force peaks of telomere G4s measured in force-ramp experiments are 17 to 25 pN in the presence of 100 mM K^+^ (loading rate 2–5.5 pN/s) [52,61] and 5 to 15 pN in the presence of 100 mM Na^+^ [79,98], which corresponds to a zero-force unfolding rate in the range of 10^−3^ to 10^−2^ s^−1^.

Single-molecule force spectroscopy enables the characterization of potential folding intermediates of telomeric G4s. In 2012, three tandem guanine repeats of human telomeric DNA were investigated using OT, and species with high mechanical stability (similar to G4s) were detected [101]. The results suggest that the triplex could be stable G4s intermediates. The effects of molecular crowding and ion types on the mechanical stability of G-triplexes were also measured by OT [102]. Long-loop misfolded population has also been observed for telomeric sequences containing more than 4 TTAGGG repeats, thus suggests complicate folding pathway of the telomere G4s [103]. In 2019, the Ha groups combined smFRET and OT to measure the mechanical stability of telomeric G4s, and their result suggested the coexistence of six mechanically distinct structures (unfolding rates in the range of 10^−5^ to 10^−1^ s^−1^) of telomeric G4s in K^+^ solution [79]. The actual intermediates structures of telomeric G4s are still needed further investigation.

In contrast to telomeric G4s, sequences with G3NG3 motifs are enriched in oncogene promoters, such as c-Myc, Bcl-2, RET, VEGF, and C-KIT. These sequences prefer to form parallel-stranded G4s. In 2015, You et al. investigated the stability of the G4s formed on the promoter region of oncogene c-Myc in a physiologically relevant 100 mM K^+^ solution condition using magnetic tweezers. It has been challenging to directly obtain such knowledge in previous bulk studies due to a high stability of c-MYC G4s. The wild-type Pu27 sequence could fold into multiple G4s, and the major species had a very high mechanical stability (unfolding force peak~54 pN), which suggests a slow unfolding rate of 1.4 × 10^−6^ s^−1^ at zero force [94]. The force-dependent thermodynamics and kinetic properties of a truncated variant Myc-2345 represent the major species of the wild types measured [94]. Subsequently, the folding/unfolding kinetics of two major G4s species at the promoter of oncogene BCL-2, Bcl2-2345 and Bcl2-1245, which have distinct topologies, have been studied [104]. The parallel-stranded Bcl2-1245 G4s also exhibited high mechanical stability compared with the hybrid-stranded Bcl2-2345 G4s. Moreover, other parallel-stranded promoter G4s, such as human telomerase reverse transcriptase (hTERT) promoter G4s, also revealed a high unfolding force peak~40 pN [53].

Based on the above results, Cheng et al. systematically measured the unfolding force distributions of 22 G4-forming sequences that predominantly adopt parallel-stranded G4 structures, including 4 oncogene promoter G4s and 12 model sequences [98]. The major G4s species formed by these sequences all revealed high mechanical stability (unfolding force peak at 40–60 pN), suggesting that slow unfolding rates (in the region of 10^−7^ to 10^−5^ s^−1^) are prevalent in parallel-stranded DNA G4s (Figure 3F). In contrast, nonparallel G4s such as thrombin binding aptamer (TBA) G4s, Bcl2-2345, and telomeric G4s revealed a major unfolding force peak of <40 pN [98]. The results are consistent with other single-molecule force spectroscopy studies on nonparallel TBA G4s [105] and telomeric G4s in Na^+^ and K^+^ buffers [79]. The loop lengths decrease the folding probability and folding rates of G4s. In 2021, Zhang et al. further characterized the mechanical stability of noncanonical parallel-stranded bulged G4s. The results showed that bulged G4s form multiple conformations, including fully folded G4s with an unfolding force peak at >40 pN and partially folded intermediates (guanine-vacancy bearing G4s) with an unfolding force peak at <40 pN [62]. The effects of bulge lengths, bulge positions, and G-vacancies on the folding rates and folding probability have also been systematically analyzed [62].

In contrast to DNA G4s, the RNA G4s predominantly adopt parallel-stranded G4 conformations. However, the multiple conformations have also been found for RNA G4s. An OT study showed that RNA G4s formed on individual human telomeric RNA (TERRA) form multiple conformations with unfolding force peaks at 22 and 38 pN and different step sizes (5.5 nm and 9.3 nm) [83]. Long noncoding telomeric RNA (TERRA) [82,106] and human telomerase RNA (hTR) [107] can also form multiple G4s.

In this section, we summarized the principles and current progress of using single-molecule force spectroscopy to study the folding/unfolding dynamics and polymorphism of G4s. In 2011, Koirala et al. showed that telomere G4s folding rates can be promoted by small-molecule ligands, such as pyridostatin (PDS) and L2H2-6OTD [52]. The binding of PDS to telomere G4s resulted in the shift of the unfolding force peak of G4s in K^+^ solution from ~21 pN to~41 pN, suggesting a strong reduction in unfolding rate. Since then, the applications of force–spectroscopy techniques further enable the characterization of interactions between quadruplexes and quadruplexes [108], small ligands and quadruplexes [52,109,110,111,112], proteins and quadruplexes [63,64,65], and study on factors that affect the folding/unfolding dynamics of G4s, including molecular crowding [113], force at different directions [84,110], nanoconfinement [91,92,93], DNA superhelicity [88], and RNA transcripts [114]. The force spectroscopy has also been used to study the folding/unfolding dynamics of other four strands nucleic acid structures i-motif, which are located at the opposite strand of G4s [115,116], and the molecular switch between G4s and i-motif [117].

Among the factors that affect the dynamics of G4s, the protein-quadruplex interactions had the most significant biological relevance but were much less understood. In cells, the folding/unfolding dynamics of G4s are regulated by binding proteins. In particular, motor proteins such as polymerases or helicases may exert forces on G4s, thus studies of the force-dependent G4s dynamics and force-dependent activity of motor proteins are crucial for understanding the processes involving protein-quadruplex interactions. In the next section, we will describe the significance of G4 binding proteins and the interactions between proteins and G4s studied by single-molecule force spectroscopy.

## 3. Single-Molecule Force Spectroscopy Reveals the Interactions between Proteins and G4s

Identification of proteins that specifically bind to G4s and regulate the folding and unfolding of G4s provided insights into the existence and functions of G4s in vivo [2,118]. Recently, the Balasubramanian group used the chemical crosslink method to systematically investigate G4 binding proteins in native chromatin in cells, and their results suggest that a wide range of proteins are involved in G4 regulations [119]. Subsequently, Pipier et al. used various RNA or DNA G4s as baits in a pull-down assay of human cell extracts followed by MS-based quantitative proteomics to identify the topology-specific G4 binding proteins [120]. Using genome-wide shRNA silencing in the presence of G4-stabilizing ligands, the Balasubramanian group also systematically identified G4-associated genes/pathways based on enhanced cancer cells sensitivity to G4 ligands [121]. This result also suggests that studies on proteins and G4 interactions provide important insights for using G4s as therapeutic targets.

**Figure 4:**
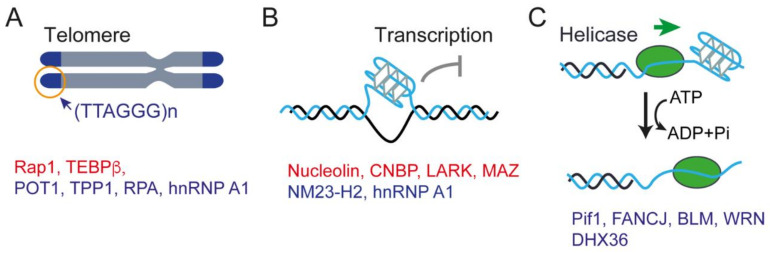
G4-interacting proteins. (**A**) Telomere and its associated proteins. (**B**) Transcription-related proteins. (**C**) Helicases. Proteins that promote G4s or stabilize G4s are shown in red. Proteins that unwind or destabilize G4s are shown in blue.

The interactions between G4s and many proteins have been studied, and Table 1 illustrates the representatives of telomere-associated proteins, helicases [122], and transcription-related proteins [11] that support the in vivo functions of G4s (Figure 4). Telomere elongation by telomerase is regulated by the shelterin complex that specifically binds to telomeric DNA. Proteins involved in shelterin complexes such as RAP1, POT, and TPP1 are shown to be involved in regulating the G4s folding/unfolding dynamics at the telomere region (Table 1 and Figure 4A). The formation of G4s also has been shown to affect the association of shelterin subunits and has an important role in the replication of telomere [123]. A total of >60% of promoters containing potential G4-forming sequences suggest the involvement of G4s in transcription regulation [10,124]. The transcriptional role of G4-interacting proteins at promoter regions including promotion and stabilization of G4s (e.g., nucleolin, CNBP, LARK, and MAZ) or unwinding of G4s (e.g., NM23-H2, hnRNP A1), therefore modulating the activation or silencing of the gene (Figure 4B). G4 formation may obstruct DNA replication; thus, G4 formation regulated by proteins such as RPA and FANCJ helicase at the replication fork has an important role in maintaining genome stability [125]. Intriguingly, a SARS unique macrodomain (SUD) within the Nsp3 protein of both SARS-CoV and SARS-CoV-2 also can bind G4 structures, thus suggesting the potential roles of G4s in viral replication and transcription [126,127].

The role of G4s as regulatory elements in telomere, promoter, and RNA regions requires that their folding and unfolding (unwinding) is temporally controlled [128]. The G4 binding proteins can accelerate the folding rate (*k*_f_) of intramolecular G4s or stabilize the folded G4s by reducing the unfolding rates (*k*_u_). Hence, in this section, we classify G4-interacting proteins in four groups: (I) G4 resolving/unwinding proteins: these proteins can change the G4s stability by accelerating the unfolding rate *k*_u_ of G4s and unwinding G4s. (II) G4 chaperones that can accelerate the folding rate *k*_f_ of G4s. (III) G4 stabilizer that can reduce the unfolding rate *k*_u_ of G4s. (IV) DNA and RNA polymerases do not directly bind to G4s, but the activity of polymerase can be affected by G4s [129], so we will also briefly introduce the effects of G4s on polymerases. Recently, it has been shown that telomere-specific DNA polymerase, the telomerase, can bind and unwind parallel-stranded G4s [73]. Single-molecule methods including fluorescence-based methods and force-based methods allow direct measurements of the folding rate *k*_f_ and unfolding rate *k*_u_ of intramolecular G4s and the extension activity of polymerase at physiologically relevant solution conditions in real time, thus providing a detailed mechanism of the interactions between proteins and G4s [41,43,44,63,64,65].

**Table 1:** Representative G4-interacting proteins.

Function	Proteins ^1^	Bind G4s	Promote G4s	Stabilize G4s	Unwind G4s	Structure ^2^ (PDB)	Reference
Telomere	Telomerase ^3^	**✓**			**✓**		[73,130]
	RAP1	**✓**	**✓**			6LDM	[131,132,133]
	TEBP-β	**✓**	**✓**			1JB7	[128,134,135,136]
	POT1-TPP1	**✓**			**✓**		[69,137,138,139]
	RPA	**✓**			**✓**		[69,123,140]
	ATRX	**✓**					[141]
	hnRNP A1	**✓**			**✓**		[142,143]
	hnRNP A2	**✓**			**✓**		[144,145]
Transcription	Nucleolin	**✓**	**✓**	**✓**			[146,147]
	NM23-H2	**✓**			**✓**		[148]
	CNBP	**✓**	**✓**				[149]
	LARK	**✓**	**✓**				[150]
	MAZ	**✓**		**✓**			[151]
	hnRNP A1	**✓**			**✓**		[143]
	YY1	**✓**					[152]
DNA repair	PARP1	**✓**					[153,154]
Replication	RPA	**✓**			**✓**		[125]
Helicase	Pif1	**✓**			**✓**		[155]
	FANCJ	**✓**			**✓**		[72,125,156]
	BLM	**✓**			**✓**		[157]
	WRN	**✓**			**✓**		[158,159,160]
	DHX36 (RHAU)	**✓**		**✓**	**✓**	5VHE	[63,161,162]
Viral protein	HIV-1 NCp	**✓**	**✓**				[163]
	Nsp3	**✓**					[126,127]
Others	Topo I	**✓**	**✓**				[164]
	BG4 antibody	**✓**	**✓**	**✓**			[64]
	thrombin	**✓**		**✓**		1HAO	[105,165,166]

RAP1, repressor activator protein 1; TEBP, telomere end-binding protein; POT1-TPP1, protection of telomeres 1-telomere protection protein 1; RPA, replication protein A; ATRX, alpha-thalassemia/mental retardation syndrome X-linked; hnRNP, heterogeneous nuclear ribonucleoprotein; CNBP, cellular nucleic-acid-binding protein; MAZ, myc-associated zinc-finger; YY1, Yin and Yang 1; PARP1, poly [ADP-ribose] polymerase 1; FANCJ, Fanconi anemia complementation group J protein; BLM, Bloom syndrome protein; WRN, Werner syndrome protein; DHX36, DEAH-box protein 36; RHAU, RNA helicase associated with AU-rich element; HIV-1 NCp, HIV-1 nucleocapsid proteins; Nsp3, non-structural protein; Topo I, topoisomerase I. ^1^ Many G4-interacting proteins are listed in the G4IPDB database [167]. ^2^ Only protein crystal structures in a complex with G-quadruplexes are presented. ^3^ Human telomerase only resolves parallel-stranded G4s but not nonparallel-stranded G4s [73].

### 3.1. G4 Resolving/Unwinding Proteins

As a result of the slow unfolding rates of G4s, eukaryotic cells encode specialized helicases to resolve or unwind the G4 structures using the energy from ATP hydrolysis [122,168]. Among them, the representative superfamily 1 Pif1 helicase [155] and superfamily 2 helicases Fanconi anemia complementation group J (FANCJ) [125,156], Bloom syndrome protein (BLM) [157], Werner syndrome protein (WRN) [158], and DEAH-box helicase DHX36 (also known as RHAU or G4R1) [161] are the best characterized G4 resolvases. The presence of G4s in vivo can act as a roadblock to affect cell processes, such as replication procession and double-strand breaks, therefore causing genome instability (Figure 4C) [155]. Cells express different types of helicases probably related to the processing of various G4 conformations. Hence, studies on the interactions between G4s and helicases from a single-molecule perspective are essential for understanding the molecular mechanism of the recognition and unwinding of G4s by specific helicases.

Unlike helicases that unwinding G4 structures require for ATP, several single-stranded DNA binding proteins such as POT1 and RPA have also been shown to resolve G4 structures in the absence of ATP in vitro. POT1 binds to the 3′-overhang of telomeric repeats and is involved in a sheltering complex that protects telomeres. RPA is involved in telomere replication and DNA replication, repair, and recombination. Using an in vitro fluorescent assay, Salas et al. demonstrated that human RPA can promote the unfolding of human telomeric DNA G4s much faster than the complementary strand [140]. Mutations of RPA affecting its binding ability to G4s can impair lagging-strand telomere replication, thus suggesting that the resolving of G4 structures by RPA has an essential role in telomere maintenance [123].

The working mechanisms of G4-specific helicases, such as Pif1 [70,71], FANCJ [72], BLM [74,75,76], WRN [76], and DHX36 (RHAU) [76,77] helicases, have been studied at the single-molecule level using smFRET. These smFRET experiments revealed the multiple steps during the unwinding of G4s, the ATP dependence of the helicase activity, and the substrate specificity of helicases. The resolution of POT1-TPP1 and RPA-mediated G4s has also been investigated by smFRET, which provides important insights for the interactions and regulatory functions of these proteins [69]. However, whether these proteins actively destabilize G4s or simply trap the unfolded ssDNA remains elusive and undetermined. Compared to biochemical assay and fluorescent-based single-molecule methods, force-based methods can provide force-dependent unwinding activity of helicases and direct measurement on the stability of G4s regulated by proteins.

**Figure 5:**
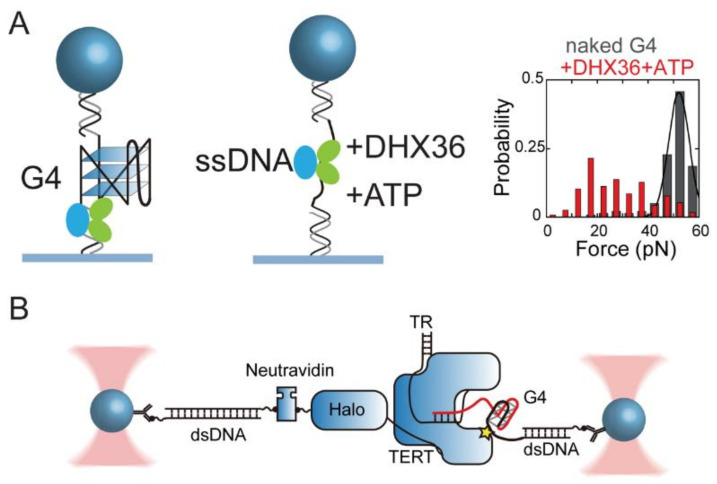
Example applications of force spectroscopy to study the proteins and G4 interactions. (**A**) Experimental design to measure the interaction between DHX36 (RHAU) helicase and c-Myc DNA G4 by MT. Adapted with permission from [63], Oxford Academic. (**B**) Experimental design to measure the telomerase elongation processivity by OT. Telomerase and its substrate were attached to two polystyrene beads through dsDNA handles. Adapted with permission from [65], Springer Nature Limited.

Using single-molecule MT, You et al. investigated the binding of DHX36 helicase to G4 structures at different intermediate states during the DHX36 ATPase cycle (Figure 5A) [63]. By detecting the lifetime of G4s under tension in force-jump measurements, the results showed that DHX36 helicase binds to a G4 structure in a nucleotide-free, AMP-PNP (non-hydrolysable ATP analogue), and ADP state resulting in G4 stabilization. The ADP AlF4-bound DHX36, which mimics the ATP hydrolysis intermediate state, significantly decreases the lifetime of G4s thus suggesting the strong G4 destabilization effects. By combining the use of truncated mutant of DHX36 and deletion of the 3′ ssDNA binding tail, the role of the DHX36-specific motif (RSM) and the 3′ ssDNA tail in the recognition and unwinding activity of DHX36 helicase has also been analyzed. These results are consistent with the structure of DHX36 helicase at different ATP hydrolysis intermediate states [169]. The co-crystallized structure of DHX36 bound with G4 DNA revealed a “register-shifted” G4 with 5′-tetrad composed of noncanonical AT-G-G-quartet structure, which might correspond to partially destabilized G4s upon binding by DHX36 [162].

### 3.2. G4 Chaperone and G4 Stabilizer

An early in vitro gel mobility shift assay and CD experiments show that telomere end-binding protein β (TEBPβ) from *Oxytricha* [134] and telomeric protein Rap1 from *S. cerevisiae* [131] can promote the tetramolecular G4 formation in a molecular chaperone-like manner. Rap 1 can also promote intramolecular parallel-stranded G4 formation in the absence of K^+^ [132]. In particular, the regulation of the formation of G4 in telomere in ciliates during the cell cycle has been further proved by in vivo studies [136]. Many G4-promoting or stabilizing proteins are involved in transcriptional regulation (Table 1), such as nucleolin [146], cellular nucleic acid-binding protein (CNBP) [170], LARK [150], and myc-associated zinc-finger (MAZ) [151]. Interestingly, retroviral HIV-1 nucleocapsid proteins also have been shown to act as a nucleic acid chaperones in promoting G4s formation [163]. However, the promotion of intramolecular G4s formation is often characterized in vitro by the absence of K^+^ but the presence of G4s chaperone proteins, which is not a physiological buffer condition. The stabilization of G4s by proteins is often characterized by melting curve measurements; however, many G4s with high melting temperatures *T*_m_ (e.g., c-Myc G4) cannot be measured at a physiologically relevant K^+^ concentration. How the folding/unfolding kinetics of G4s are affected by these binding proteins under physiologically relevant buffer conditions remain largely unknown.

Using single-molecule force spectroscopy, the effects of proteins on the folding and unfolding rates of intramolecular G4s at physiologically relevant conditions can be directly characterized. For instance, Yangyuoru et al. analyzed the effects of binding proteins on the *k*_f_ and *k*_u_ of intramolecular G4s in the presence of 100 mM K^+^ using force spectroscopy [64]. A TERRA G4 sequence (5′-UUA(GGGUUA)_4_-3′) is spanned by two DNA–RNA hybrid handles attached to two beads in OT. The folding rate of TERRA RNA G4 is measured in the presence of G4 selective antibody BG4 based on measuring the time-evolution of the folding probability *p*_fold_(t). The *k*_f_ of TERRA G4s in the presence of BG4 (7.5 × 10^−2^ s^−1^) was two-fold faster than in the absence of antibody or G4s ligands (3.3 × 10^−2^ s^−1^). In the presence of 50 nM BG4, the unfolding force peaks of TERRA G4s measured in force-ramp experiments increased from 23 and 36 pN to 24 pN and 40 pN, suggesting that BG4 increases the mechanical stability and reduces the unfolding rate *k*_u_ of the TERRA G4s. The similar experimental setup and principle can be applied to a wide range of G4-interacting proteins.

### 3.3. The Effects of G4s on DNA and RNA Polymerases

G4s function as a molecular knot that presents an obstacle to DNA and RNA polymerases. On the other hand, the formation of G4s might be involved in the activation of some DNA replication origins as well as in recruiting transcription factors to promoters and enhancing the expression of mRNA [171,172]. The interactions between telomere-specific polymerase, the telomerase, and the G-quadruplexes formed by telomeric DNA have received the most attention. In 1991, Zahler et al. showed that folding of telomeric DNA into G4s inhibits the extension of telomerase [129]. Small ligand stabilizing G4s can further inhibit telomerase and thus have anti-cancer activity [173]. Recently, however, this view has been challenged by recent findings that show that telomerase can unfold and extend parallel-stranded G4s but not nonparallel G4s using smFRET and bulk assays [73,130]. Jansson et al. used single-molecule fluorescence combined with biochemical methods and showed that G4s can be formed during the synthesis of telomeric DNA products by telomerase, and the G4 formation participated in the catalysis and dissociation process of the telomerase [174]. These findings suggest that G4s not only inhibit the telomerase but play a role in regulating the activity of the telomerase, and the folding topologies G4s are important for the regulatory functions.

During nuclear assembly, the human telomeres are not freely diffused but tethered to the nuclear envelope [175]. The mechanical forces on the tethered chromosome ends may also regulate the dynamics of telomere structure and the activity of telomerase. Owing to the difficulty in reconstituting a large active telomerase complex using single-molecule force spectroscopy, the force-dependent activity of telomerase was lacking.

A breakthrough of using force-based measurements on the activity of telomerase was reported in 2020. Patrick et al. used high-resolution dual-trap optical tweezers to study the telomerase extension during the processive extension by telomerase (Figure 5B) [65]. A telomerase and its substrate DNA are connected to two anti-digoxin-coated polystyrene beads separately through two dsDNA handles labeled with digoxigenin. By applying a constant force (4.0–4.5 pN) to the DNA tethers and transferring the tether to buffer with dNTP, telomerase synthesizes telomeric repeats and the telomerase extension activity can be measured based on the distance between the two beads. Based on the processive elongation, and forward and reverse stepping in the time trajectories, they demonstrated that telomerase synthesizes multiple telomeric repeats before being released from the substrate; they also provided evidence that the interactions between the substrate DNA and an anchor site of telomerase prevent the dissociation of telomerase from substrate during translocation. Moreover, they showed that a dynamic folding/unfolding transition of G4s contributes to the small forward reverse step (15 nt) in trajectories, thus suggesting that the formation of G4s plays an important role in recapturing the telomerase to the DNA substrate. This experiment allowed direct quantification of the steps of telomere elongation, the time constants of a single telomerase associated with DNA substrates, and the folding/unfolding dynamics of G4s, thereby furthering our understanding on the working mechanisms of telomerase.

## 4. Conclusions and Future Perspectives

In summary, this review introduced the basic biophysical principles and recent progress of using single-molecule force spectroscopy to study the folding/unfolding dynamics of G4 nucleic acid structures and the interactions with proteins. These results provided compelling new data on the regulatory functions of the G4s. With the benefit of single-molecule force spectroscopy, the transition kinetics of individual G4 structures in a heterogeneous sample and complicated interconversion among different conformations can be resolved. The effects of ion conditions, molecular crowding, small ligands, loop length, G-vacancy on the stability and dynamics of G4s have been gradually uncovered [41]. In addition, the molecular mechanism of helicase unwinding G4s [63], the processive telomerase extension [65], and BG4 antibody effects on the folding/unfolding dynamics of TERRA G4s [64] have been elucidated in detail using single-molecule force spectroscopy. These findings provide new insights for better understanding the roles of G4s in vivo.

A systematic analysis showed that an increasing number of proteins [119,120] and genes [121] in human cells interact with G4s or are involved in the pathways regulating G4s. The shRNA silencing of genes can sensitize G4 ligand-induced cancer cell death, thus suggesting the significance of the characterization of protein–G4s interactions for G4-based therapies [121]. Studies on promoter G4s showed that a G4s specific small ligand quarfloxin (CX-3543) inhibits Pol I transcription through disruption of the G4s–nucleolin complex, thus suggesting that the protein–G4s complexes are the target that leads to the biological consequences [176]. Therefore, a further challenge for targeting G4s includes characterization of the complex interactions between small ligands, proteins, and G4s at the single-molecule level [15,16].

Despite the intensive investigation of the G4-interacting proteins, the molecular mechanisms for the recognition and stabilization or destabilization of G4s by proteins remains poorly understood [39,177]. Until now, only limited numbers of high-resolution crystallographic and NMR structures of the protein–G4s complex have been resolved [133,135,162,177]. The structure of RAP1 and DHX36 suggest that the interaction of an α-helix with a G-tetrad is a conserved motif that can be used to recognize G4 structures [133,162]. In contrast to the DHX36-specific motif that interacts with the planar G-tetrad, many of the G4 binding proteins have an RGG domain, such as nucleolin, which interacts mainly with the loop region of G4s [177]. However, for most of the G4-interacting proteins, how the specific interactions with G4s stabilize or destabilize G4 structures remain largely unknown. For proteins such as DHX36, the cooperative interactions through the OB-fold domain and a flexible loop are also important for the unwinding activity of G4s, as suggested by single-molecule force spectroscopy [63] and a recent molecular dynamic simulation [178]. Taken together, single-molecule techniques together with other emerging techniques including molecular dynamics simulations and quantum mechanics methods [38,39], smFRET [42,43], and single-molecule visualization of G4 dynamics in live cells [78] are an inevitable trend in future research and can demystify the specific interactions that regulate the dynamics and stability of G4s in vitro and in vivo.
